# Recent advances in reactive oxygen species scavenging nanomaterials for wound healing

**DOI:** 10.1002/EXP.20230066

**Published:** 2024-01-17

**Authors:** Alireza Joorabloo, Tianqing Liu

**Affiliations:** ^1^ NICM Health Research Institute Western Sydney University Westmead Australia

**Keywords:** antioxidative nanomaterials, oxidative stress, reactive oxygen species, wound healing

## Abstract

Reactive oxygen species play a crucial role in cell signaling pathways during wound healing phases. Treatment strategies to balance the redox level in the deep wound tissue are emerging for wound management. In recent years, reactive oxygen species scavenging agents including natural antioxidants, reactive oxygen species (ROS) scavenging nanozymes, and antioxidant delivery systems have been widely employed to inhibit oxidative stress and promote skin regeneration. Here, the importance of reactive oxygen species in different wound healing phases is critically analyzed. Various cutting‐edge bioactive ROS nanoscavengers and antioxidant delivery platforms are discussed. This review also highlights the future directions for wound therapies via reactive oxygen species scavenging. This comprehensive review offers a map of the research on ROS scavengers with redox balancing mechanisms of action in the wound healing process, which benefits development and clinical applications of next‐generation ROS scavenging‐based nanomaterials in skin regeneration.

## Graphical abstract

Here, the essential role of redox balance in wound healing and skin regeneration is summarized. Reactive oxygen species (ROS) scavenging strategies are classified and discussed along with the most recent work. In addition, valuable insights are offered for future research directions and is expected to promote further studies on the ROS scavenging nanomaterials for wound healing.

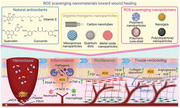

## 1 INTRODUCTION

Affecting more than 20 million worldwide, wound treatment has a significant economic impact on health care.^[^ ^1^ ^]^ Although normal wound healing is a highly dynamic and well organized process including hemostasis, inflammation, proliferation, and tissue remodeling phases, this healing process is normally impaired by aging and chronic conditions. Chronic wounds, such as diabetic ulcers and pressure sores, takes several months or never heal due to persistent on inflammation, imbalance of tissue regeneration and degradation, and infection or biofilm formation.^[^ ^2^, ^3^, ^4^, ^5^ ^]^ They are also associated with complications, such as infection, osteomyelitis, tissue necrosis, hematomas, and even mental health issues (e.g. anxiety and depression). Therefore, wound management remains a challenge in the clinic.

Reactive oxygen species (ROS), such as superoxide anion (O_2_^‒•^), hydroxyl radicals (OH^•^), peroxide (O_2_^2–^), and hydrogen peroxide (H_2_O_2_), play an important role in intracellular signaling for wound healing and infection. When the pathogens caused the infection of the wound, phagocytic cells produce cytokines and ROS to provide an antimicrobial state and enhance host defenses to accelerate the removal of pathogens and wound debris.^[^ ^6^, ^7^ ^]^ Although ROS has beneficial effects as a defense mechanism against pathogens, imbalance redox and increased release of related metal ions can further induce tissue oxidative stress damage, impair cell proliferation, and cause cell death, leading to the pathological conditions such as fibrotic scarring and inflammation. Therefore, treatment strategies to balance the redox level in the deep wound tissue are emerging for wound management.

Antioxidant therapies have been considered as promising strategies in chronic wound healing given their central role in downregulating ROS and suppressing oxidative stress.^[^ ^11^, ^12^ ^]^ There are strong evidences that show the beneficial roles of naturally derived antioxidants in wound healing applications by efficiently promoting different phases of wound healing.^[^ ^2^, ^12^, ^13^ ^]^ Despite to the rapid development in the field of antioxidant therapies for wound healing, natural antioxidant compounds showed some issues during clinical applications, such as rapid clearance from the lesions, low bioavailability, and enhanced side effects due to accumulation. Therefore, there is an urgent need to develop advanced therapies to treat oxidative stress and restore redox hemostasis in skin regeneration.^[^ ^11^ ^]^

The novel treatment strategies to balance ROS levels in regenerative medicine and resolve inflammation have been introduced, such as ROS scavenging enzyme‐mimetic nanomaterials, nanomaterials combined with antioxidants, and ROS responsive nanomaterials and polymeric nanoparticles.^[^ ^8^, ^9^, ^10^ ^]^ Nanomaterials with ROS scavenging ability have shown great potentials to remove excessive free radicals and restore redox balance to the normal level during wound healing. This novel antioxidant therapeutic strategy can either mimic the enzymatic activities to neutralize ROS or deliver antioxidant agents to improve anti‐inflammatory therapeutic outcomes around the wound regions.^[^ ^14^, ^15^, ^16^, ^17^, ^18^ ^]^ Moreover, these ROS nanoscavengers can be engineered with outstanding characteristics, including robust catalytic activities, high enzymatic stability under physiological conditions, large surface area, easy large‐scale production and low cost, and surface modification possibilities, which can benefit the pharmaceutical performance of the antioxidant therapy.^[^ ^19^, ^20^ ^]^ In this review, we focus on the various ROS scavenging nanomaterials, such as natural antioxidants and nanoenzymes, organic/inorganic‐based nanoparticles, and ROS‐responsive polymeric nanomaterials in the treatment of wounds.

## 2 REDOX HOMEOSTASIS IN WOUND HEALING PROCESSES

**FIGURE 1:**
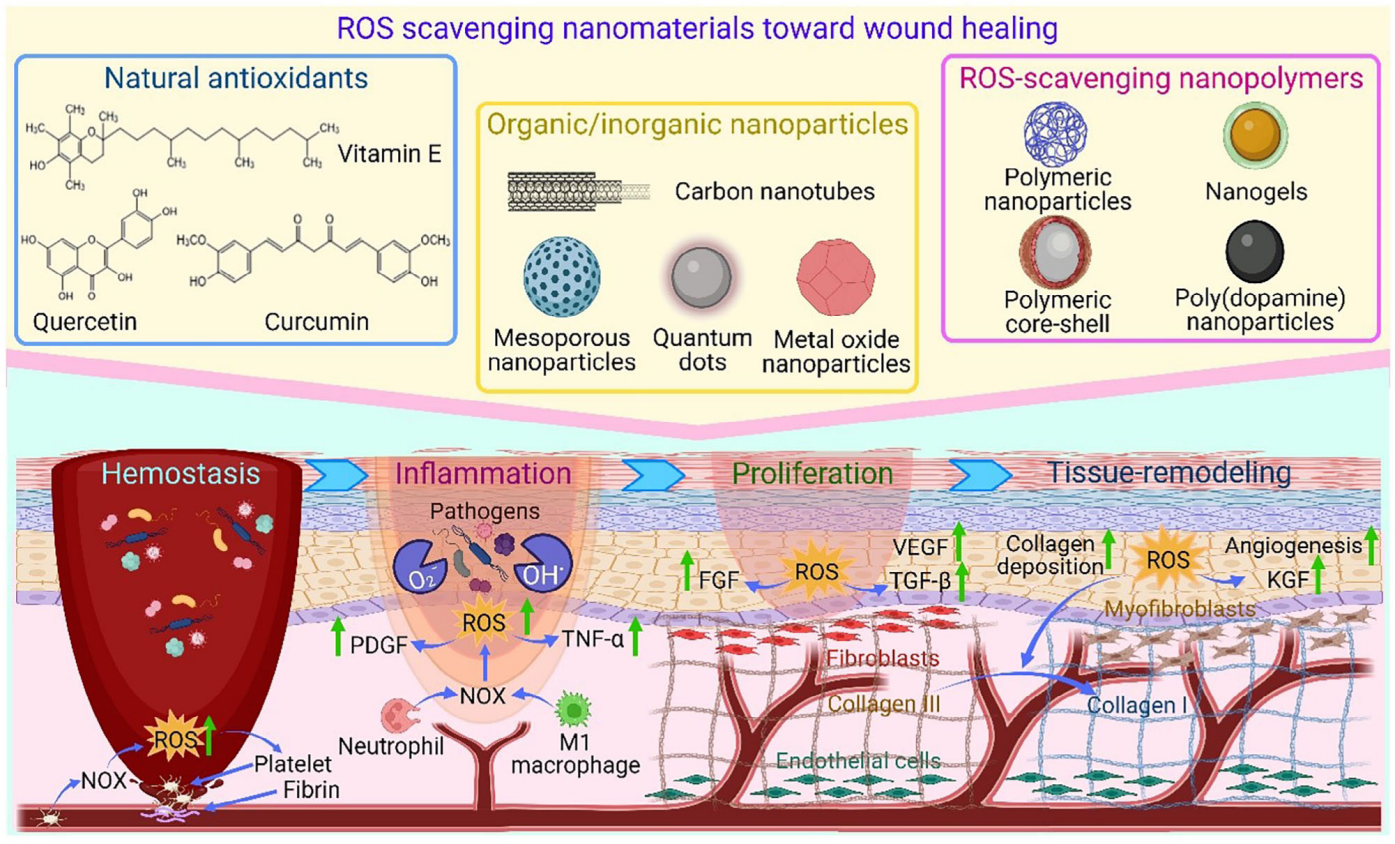
The effect of different types of reactive oxygen species (ROS) scavenging nanomaterials on wound healing process with the involvement of the key cellular populations in the four healing phases.

Serving as cell signaling messengers, ROS play vital roles in the wound healing process.^[^ ^2^, ^20^ ^]^ They are generally produced by the respiratory chain in mitochondria and are mainly compromised of various oxidant molecules such as H_2_O_2_, O_2_^–•^, O_2_^2–^, or OH^•^.^[^ ^2^, ^7^, ^21^, ^22^, ^23^ ^]^ In homeostasis phase, ROS are produced by NADPH oxidases (NOX) enzymes in vascular cells as a result of secreted factors by platelets to stimulate chemotaxis and adhesion molecule expression and consequently reduce local blood flow via vasoconstriction and thrombus formation.^[^ ^4^, ^24^ ^]^ During inflammation phase, a high level of produced superoxide and H_2_O_2_ by neutrophils and macrophages via NOX plays a crucial role in bacterial killing and prevention of wound infection. ROS also have the ability to stimulate tumor necrosis factor‐α (TNF‐α) and platelet‐derived growth factor (PDGF) release and support migration of monocytes and macrophages to the wound sites to invade pathogens.^[^ ^4^, ^22^, ^24^, ^25^, ^26^ ^]^ Redox signaling is also required for the proliferation phase. ROS mediate tissue growth factor‐β1 (TGF‐β1) signaling to promote the expression of fibroblast growth factor (FGF), fibroblasts proliferation and migration, and synthesis and migration of collagen and fibronectin. In addition, ROS stimulate angiogenesis, endothelial cell division and migration to facilitate blood vessel formation through the expression of vascular endothelial growth factor (VEGF).^[^ ^4^, ^22^, ^24^ ^]^ In tissue remodeling phase and extracellular matrix (ECM) reconstruction, ROS promote keratinocytes growth and migration via triggering keratinocyte growth factor (KGF) receptor activation and internalization.^[^ ^4^, ^27^ ^]^ At the same time, ROS facilitate wound edge formation and differentiation of fibroblasts into myofibroblasts leading to collagen deposition and fibrosis (Figure 1).^[^ ^4^, ^28^ ^]^

Low level of oxygen stimulates the generation of ROS in mitochondria and activation of prolyl‐4 hydroxylases which can induce hypoxia‐inducible factor 1 (HIF‐1) to protect tissues from infection after injury and produce cell surviving signaling for effective wound healing.^[^ ^29^, ^30^ ^]^ However, uncontrolled production of ROS leads to oxidative stress, prolonged or chronic hypoxia, and disturbance of the immune system.^[^ ^9^, ^23^, ^29^, ^31^ ^]^

**FIGURE 2:**
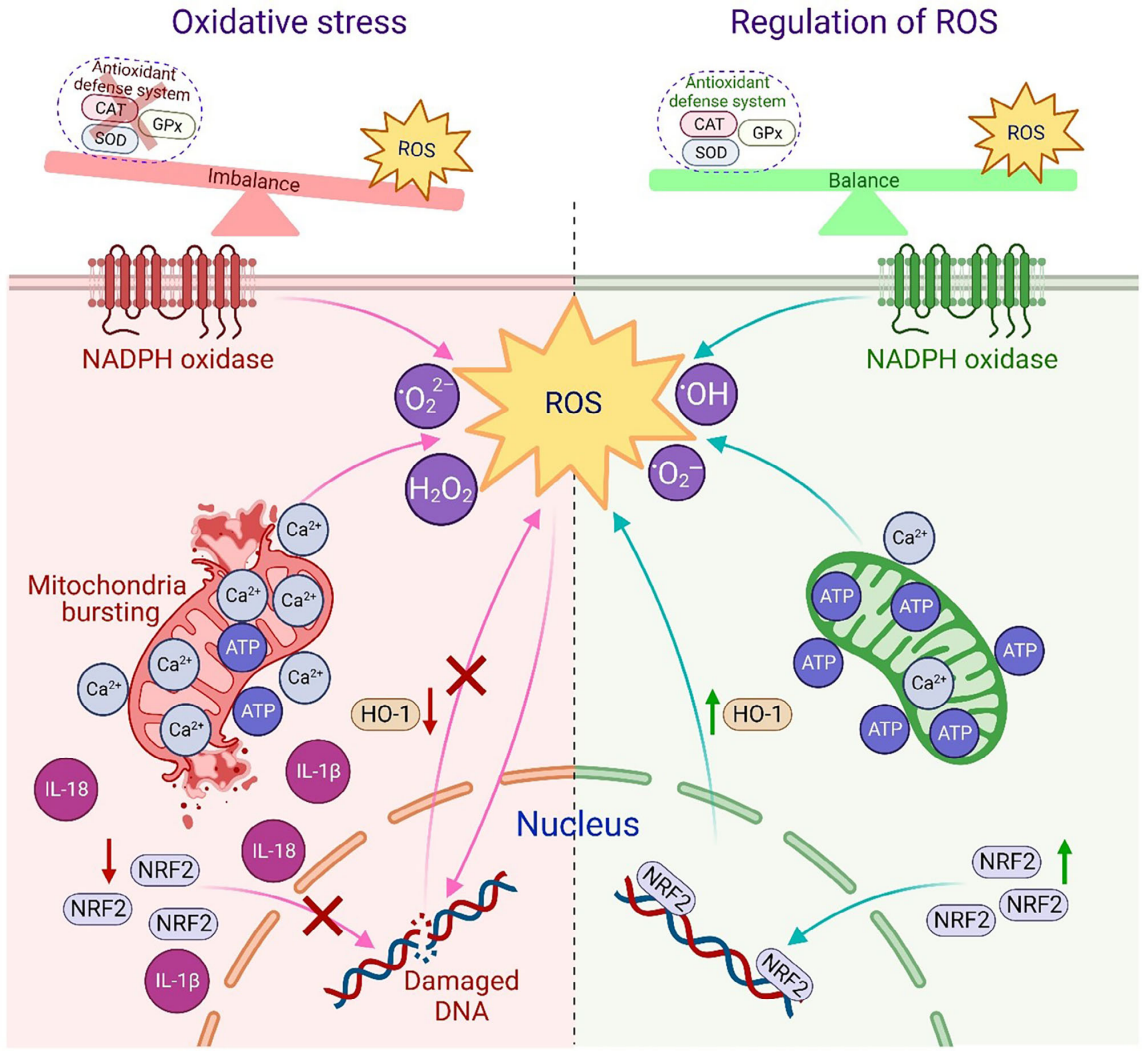
The influence of oxidative stress and regulation of reactive oxygen species (ROS) on mitochondria functionality and cell ingredients performance.

The main antioxidant enzymes including superoxide dismutase (SOD), catalase (CAT), glutathione peroxidases (GPx), and thioredoxin reductase are the key players in the antioxidant defense system.^[^ ^32^, ^33^ ^]^ The abnormal ROS production and the malfunction of the antioxidant defense system can cause cellular oxidative damages.^[^ ^32^ ^]^ Oxidative stress can impair mitochondrial integrity and cause the release of pro‐inflammatory mediators or apoptotic cell death.^[^ ^34^, ^35^, ^36^ ^]^ Excessive ROS production can induce dysregulated calcium hemostasis. Released Ca^2+^ from the endoplasmic reticulum (ER) can lead to mitochondrial Ca^2+^ overload, mitochondrial depolarization, and abnormal mitochondrial functionality.^[^ ^37^ ^]^ In addition, because the mitochondria are the primary site of adenosine triphosphate (ATP) production, the ATP level may significantly decrease within cells in the presence of oxidative stress.^[^ ^35^, ^38^ ^]^ Moreover, ROS have an impact on transcription factors such as activator protein 1 (AP‐1), nuclear factor kappa B (NF‐κB), and nuclear factor erythroid 2‐related factor 2 (NRF2) proteins in injured skin tissue.^[^ ^38^ ^]^ Among these factors, NRF2 regulates redox hemostasis and regenerative processes and heme oxygenase‐1 (HO‐1) is the primary downstream NRF2 target associated with antioxidant enzyme production.^[^ ^34^, ^39^ ^]^ NRF2 is also essential for controlling the re‐epithelialization.^[^ ^38^ ^]^ In contrast, NF‐κB and AP‐1 activation can increase matrices metalloproteins (MMPs) levels, which may cause ECM protein breakdown and slow wound healing process.^[^ ^38^ ^]^ Oxidative stress reduces NRF2 protein level in cells and inhibits NRF2/HO‐1 pathway leading to the expression of inflammatory cytokines such as interlukin‐1β (IL‐1β) and IL‐18, nucleus disruption, and DNA damages^[^ ^34^, ^38^ ^]^ (Figure 2). Therefore, a suitable balance between high and low level of ROS is essential for wound healing.

## 3 REACTIVE OXYGEN SPECIES SCAVENGING NANOMATERIALS

With the rapid development of nanotechnology, various ROS scavenging nanomaterials including metal oxide nanoparticles, carbon‐based and polymeric nanomaterials, have been engineered for wound healing. Studies have demonstrated that nanoscavengers can eliminate excessive ROS at the wound sites and suppress the oxidative stress, preventing the deepening or aggravation of wounds.^[^ ^40^, ^41^, ^42^, ^43^ ^]^ The current ROS scavenging nanomaterials and their scavenging mechanisms as well as their roles in wound healing process are discussed and summarized in Table 1.

**TABLE 1:** Nanoparticles with antioxidant activities and their influences on wound healing process.

Nanoparticles category	Shape	Nanoparticles carrier	Setting (in vitro/in vivo)	Description	Reference
Cerium oxide	Rhombohedral nanoparticle 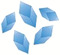	A sprayable catechol‐modified hydrogel	In vivo	CeNPs have been incorporated into a sprayable catechol‐modified hydrogel for ROS scavenging activities resulted in stimulating wound healing. ROS scavenging ability of hydrogels containing CeNPs significantly improved as well as the rate of re‐epithelialization, collagen deposition, and wound closure.	[14]
	Nanocrystal 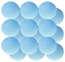	Mesoporous silica nanoparticles	In vivo	CeNPs decorated mesoporous silica nanoparticles were prepared to accelerate wound healing with ROS‐scavenging activity, tissue adhesiveness, and limited scar formation. The ROS level, H_2_O_2_ concentration, and superoxide anion rate dramatically decreased by using CeNPs decorated mesoporous silica nanoparticles.	[16]
	Nanocube 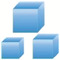	Chitosan hydrogels	In vivo	CeNPs incorporated into chitosan to determine the wound healing potential of the nanomaterials. The level of antioxidant enzymes including SOD, CAT, and GPx increased at the wound site.	[48]
	Hallow nanoparticle 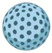	Siloxane nanoparticles and poly(vinylpyrrolidone) hydrogels	In vivo	CeNPs with hallow structure have been employed to enhance wound healing process. In proliferation phase, CeNPs captured excessive ROS to stimulate epithelial cell proliferation.	[49]
	Nanorod 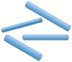	PF127 hydrogels	In vivo	CeNPs coated with poly(ethylenimine) to prepare nanocomposite hydrogels possessed ROS scavenging activity. The hydroxyl radical scavenging activity and superoxide anion inhibition rate were enhanced by increasing the concentration of cerium oxide nanorods. Relative wound area and complete wound closure time dramatically decreased in wounds treated with cerium oxide nanorods modified hydrogels.	[50]
Carbon‐based	Nanodot 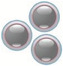	A nanofibrous poly(caprolactone)/gelatin	In vivo	ROS scavenging ability of CNDs was assessed using the expression of SOD and GPx. CNDs incorporated scaffolds with significant antioxidant activity not only reduce oxidative stress but also monitor cell/scaffold interaction to enhance wound healing via re‐epithelialization and collagen deposition.	[54]
		A topical system	In vivo	Onion derived carbon nano dots were prepared to modulate ROS level and accelerate wound healing. Increasing carbon nano dots concentration led to increased antioxidant ability and inhibition rate of superoxide.	[40]
		–	In vivo	The antioxidant activity of CNDs was explored in both the genes SOD and GPx that there is a down‐regulation in the presence of CNDs. The potential of CNDs in accelerated wound healing was observed in the histological analysis.	[55]
		Multifunctional hydrogels including methacrylated gelatin	In vivo	Antioxidant effects of CQDs in the human bone marrow mesenchymal stem cells and human dermal fibroblasts were evaluated. Wound healing efficiency of the hydrogels was evaluated via inducing the proliferation of fibroblasts, enhancing cell migration, alleviating inflammation, skin re‐epithelialization, and collagen deposition.	[56]
Iron‐based	Nanocube 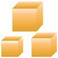	–	In vivo	Prussian blue nanoenzymes were synthesized to promote full‐thickness wounds healing. Antioxidant activity of Prussian blue nanoenzymes was measured to show the enhanced degradation rate of H_2_O_2_ and superoxide radicals through CAT and SOD mimetic activities by increasing the concentration of Prussian blue. The topical administration of Prussian blue nanoenzymes exhibited effective collagen deposition, mature organization, keratinocyte differentiation, neovascularization, and capillary formation during wound healing process.	[65]
	Nanospindle 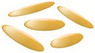	Electrospun poly(vinyl alcohol) porous scaffold	In vitro	Hematite (α‐Fe_2_O_3_) nanoenzyme particles were incorporated into electrospun poly(vinyl alcohol) porous scaffold to regulate ROS level in wound healing process. High CAT activity and quickly conversion of H_2_O_2_ to O_2_ were demonstrated to show the potential of the fabricated nanofibrous membranes as wound dressing materials.	[61]
	Nanocrystal 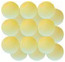	–	In vivo	IONPs were synthesized to promote peroxide‐like catalytic activity can induce conversion of H_2_O_2_ to hydroxyl radicals to prevent bacterial infection and biofilm formation and efficiently enhanced wound healing.	[60]
		A thermosensitive poly (d,l‐lactide)‐poly(ethylene glycol)‐poly(d,l‐lactide) hydrogel	In vivo	Prussian blue nanoenzyme were synthesized to promote diabetic wounds healing. The Prussian blue nanoenzymes restored mitochondrial membrane potential, reduced calcium concentration, and restored ATP production. In vivo analysis revealed the promotion of diabetic wound healing via reducing the inflammatory cytokines such as IL‐6 and TNF‐α, increasing the expression of CD31 and α‐SMA as anti‐inflammatory cytokines, neovascularization, and angiogenesis.	[34]
	Nanosphere 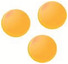	A shell of glucose oxidase	In vivo	IONPs were coated with a shell of glucose oxidase to modulate CAT and peroxide‐like activities in healing of diabetic ulcer. The elimination of the excessive oxidative stress led to short inflammatory phase, and accelerated proliferation and tissue‐remodeling phases.	[66]
Copper‐based	Irregular nanoparticle 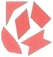	Carboxymethyl chitosan grafted glutathione hydrogels	In vivo	A hydrogel dressing containing CuNPs and carboxymethyl chitosan grafted glutathione was synthesized with dual effects of antibacterial and antioxidant. Free radical scavenging rate of superoxide and hydroxyl radicals significantly increased in hydrogels containing CuNPs.	[72]
	Nanocube 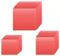	A topical system	In vivo	Ultrasmall CuNPs were synthetized with broad ROS scavenging abilities in order to tackle broad ROS‐related diseases especially diabetic wound treatment. CuNPs simultaneously possessing CAT, SOD, and glutathione peroxide mimicking enzyme properties.	[70]
	Nanosphere 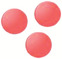	A nanocomposite hydrogel comprising a functionalized poly(ethylene glycol) and heparin	In vivo	A nanocomposite hydrogel for ROS scavenging was developed to improve inflammation inhibition and scavenging ROS to mitigate oxidative stress and promotion of angiogenesis in acute and diabetic wounds.	[73]
Molybdenum disulfide	Nanoflower 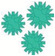	An antibacterial system based on poly(ethylene glycol)	In vivo	An antibacterial system based on poly(ethylene glycol) functionalized MoS_2_ nanoflowers was developed to induce peroxidase catalytic activity. The conversion of H_2_O_2_ into hydroxyl radicals can avoid the toxicity of high concentration of H_2_O_2_ and the hydroxyl radicals are more effective against bacteria and wounds more easily cured.	[76]
	Nanosheet 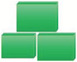	A hydrogel composed of oxidized dextran and glycol chitosan	In vivo	MoS_2_ nanosheets have been synthesized and anchored onto hydrogels to promote diabetic wound healing via reducing oxidative stress through SOD and CAT mechanisms. The O_2_® ^•^ scavenging ratio, wound closure, collagen deposition, and the expression level of CD31 and EGF increased significantly.	[80]
		A hydrogel composed of sodium alginate and poly(vinyl alcohol)	In vivo	MoS_2_ nanosheets loaded onto carbon nanotubes to exhibited SOD, CAT, and enhanced antibacterial activity aiming skin reconstruction through promoting collagen deposition and angiogenesis. The expression level of TNF‐α (an inflammatory cytokine) decreased while the expression of VEGF (an anti‐inflammatory cytokine) increased in wound treated with MoS_2_ nanosheets.	[81]
ROS‐scavenging polymers	Nanosphere 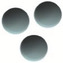	Oxidized dextran/chitosan hydrogels	In vivo	PDA nanoparticles were synthesized and incorporated into oxidized dextran/chitosan hydrogels to promote antioxidant activities and antibacterial properties for accelerated wound healing. Free radical scavenging activity of the hydrogels enhanced by increasing the concentration of PDA nanoparticles. In addition, proper skin regeneration was observed.	[15]
		A hydrogel containing poly(l‐lysine)‐grafted nanocellulose fibers and gelatin	In vivo	Antioxidant activity of the hydrogels contributed to PDA nanoparticles. The expression of IL‐6 and TNF‐α significantly decreased at the last stage of wound healing in wounds treated with NCF‐EPL/GTP/PDA hydrogels to promote cell proliferation and full‐thickness infected wound healing.	[88]
	Nanoparticle in core–shell microneedle 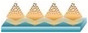	A core‐shell hyaluronic acid microneedle patch	In vivo	A core‐shell hyaluronic acid microneedle patch with ferrum‐mesenchymal stem cells in the core and PDA nanoparticles in the needle tip is prepared for wound healing. The released PDA suppresses the ROS.	[84]
	Nanoshee 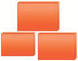	A 2D PDA nanosheet	In vivo	A 2D PDA nanosheet was prepared to act as free radical scavenger and in full‐thickness wound healing process. The histologic study shows the effectively stimulated angiogenesis and collagen deposition at the wound sites.	[85]
	Nanofiber 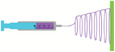	Poly(caprolactone) electrospun nanofibers	In vivo	Poly(caprolactone) electrospun nanofibers loaded with PDA and VEGF were prepared to promote wound healing of diabetic mice. Free radical scavenging property of PDA nanoparticles on the electrospun membrane was verified. In addition, enhanced wound healing through the angiogenesis and anti‐inflammatory effect of the nanofibers was observed.	[83]
		Multifunctional poly(caprolactone) nanofibers	In vitro	Multifunctional poly(caprolactone) nanofibers modified with poly(l‐lysine) and PDA was fabricated. PDA served as antioxidant agent to eliminate the excessive ROS. The electrospun nanofibers showed great potential as promising wound dressing materials.	[86]
	A layer coated on nanosphere 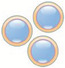	Poly(tannic acid) coated on bioactive nanoglass	In vivo	The antioxidant activity of poly(tannic acid) has been proved. Angiogenesis, promotion of proliferation, and wound treatment have been demonstrated. The expression level of IL‐6 as pro‐inflammatory cytokine decreased in wounds treated with BGN@PTE, while the expression of CD31, as a marker of vascular endothelial cells increased to show neovascularization and the efficiency of BGN@PTE in wound healing process.	[91]
	Nanorod 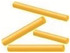	A polysaccharide matrix comprising oxidized β‐glucan and quaternized chitosan	In vivo	Poly(tannic acid) nanorods was synthesized and incorporated into a polysaccharide matrix comprising oxidized β‐glucan and quaternized chitosan to accelerate diabetic wound healing. Poly(tannic acid) nanorods as antioxidant polyphenol increased ROS scavenging rate. The combination of polysaccharide and polyphenol properties brought more options for the management of diabetic wounds.	[92]

### 3.1 Cerium oxide nanomaterials

**FIGURE 3:**
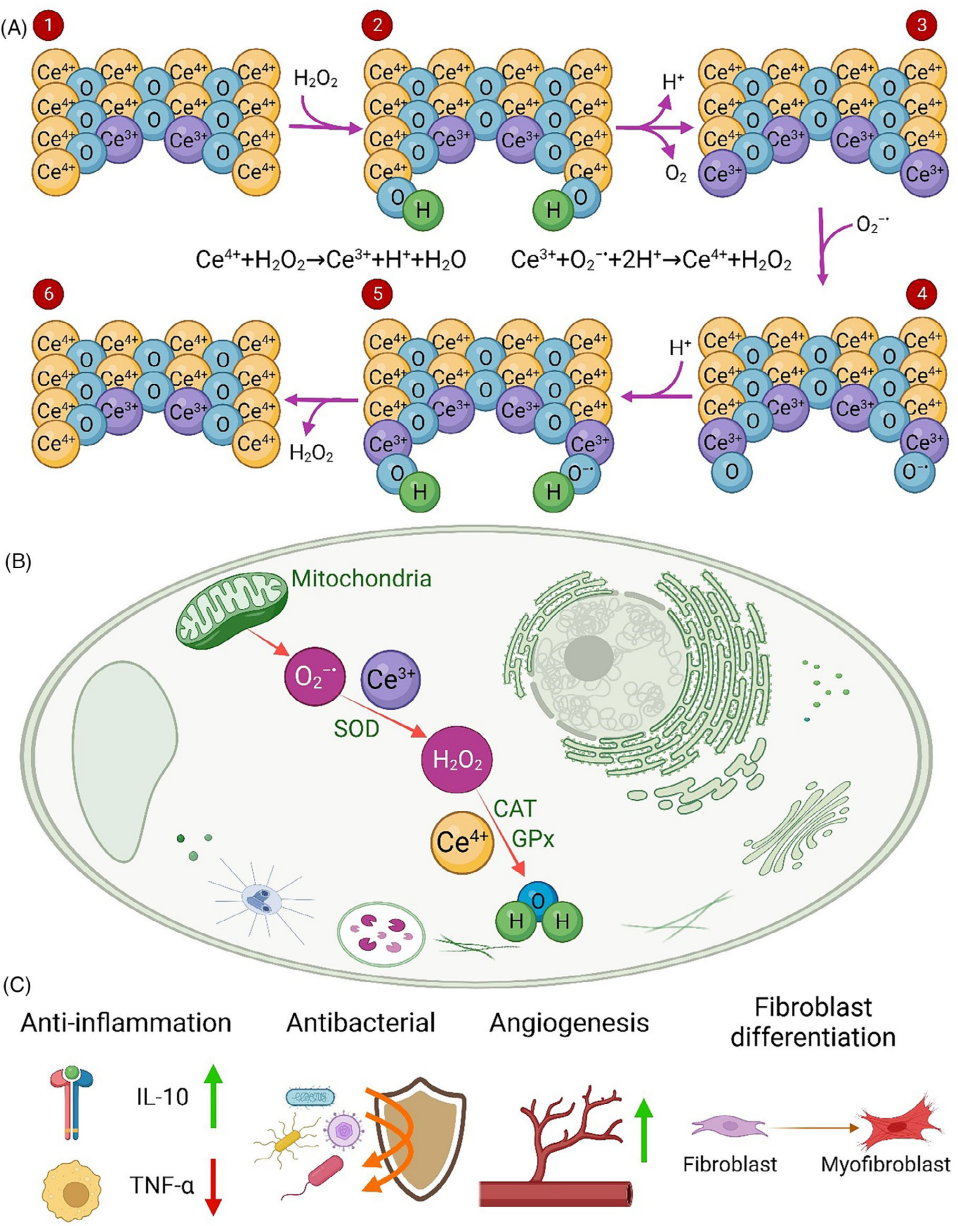
(A) Oxidation‐reduction cycle of cerium oxide that is capable to scavenge superoxide radicals and H_2_O_2_, (B) antioxidant activity of CeNPs within a cell, and (C) nanoceria therapeutic effects on wound healing process.

Cerium as a rare earth metal belongs to lanthanide series. Cerium oxide nanoparticles (nanoceria or CeNPs) are widely used as an antioxidant and oxygen or free radicals scavengers in biomedical applications.^[^ ^31^, ^44^ ^]^ Nanoceria have ROS scavenging properties via the reversible switching between Ce^3+^ (reduced) and Ce^4+^ (oxidized) states in regenerative nanoceria oxidation‐reduction cycles^[^ ^41^, ^45^, ^46^, ^47^ ^]^ as depicted in Figure 3A. Recent studies investigated the ability of nanoceria on modulation of ROS levels in skin tissue repair and regeneration^[^ ^14^, ^16^, ^48^, ^49^, ^50^ ^]^ (Figure 3B,C). Wu et al.^[^ ^16^ ^]^ fabricated ultrasmall ceria nanocrystals decorated with amino‐functionalized mesoporous silica nanoparticles (MSN) to accelerate wound healing with ROS‐scavenging activity, tissue adhesiveness, and limited scar formation. The ceria nanocrystals decorated MSN (MSN‐Ceria) showed high tissue adhesive capability compared to non‐decorated MSN. In addition, MSN‐Ceria significantly reduced oxidative stress and suppressed inflammatory response at in vivo wound sites. Histological examination demonstrated that MSN‐Ceria treated wound yielded native collagen deposition and fiber alignment compared with other groups. This MSN‐Ceria induced tissue recovery is further confirmed by the high expression of sebaceous gland marker stearoyl‐CoA desaturase 1, hair follicle stem and progenitor cells marker leucine‐rich repeats and immunoglobulin‐like domain 1, placenta‐expressed transcript‐1, and PDGF in the wound tissues, suggesting the improvement of the healed skin quality. In a study by Huang et al.,^[^ ^48^ ^]^ chitosan‐coated cerium oxide nanocubes (CCNs) were developed and their wound healing potential was determined in Sprague–Dawley rat models. The CCN treatment decreased the expression of inflammatory cytokine TNF‐α, while increased the expression of anti‐inflammatory cytokine IL‐10, indicating their anti‐inflammatory ability. They also observed that CCN‐treated wounds had compact collagen deposition, improved angiogenesis, and accelerated epithelial layer regeneration over 12 days. Also, the level of antioxidant enzymes including SOD, CAT, and GPx increased at the wound site after CCN treatment. Hollow cerium oxide nanoparticles with rough surface and l‐arginine loading (_Ah_CeO_2_ NPs) have been employed to enhance wound healing process by Ma et al.^[^ ^49^ ^]^ They demonstrated that _Ah_CeO_2_ NPs with excellent adhesion properties enabled rapid wound closure and controlled bleeding in the hemostasis phase. In addition, _Ah_CeO_2_ NPs generated abundant ROS to inhibit bacterial infections during inflammation phase, while the released l‐arginine converted into nitric oxide to stimulate epithelial cell proliferation in proliferation phase. This study showed the structure of _Ah_CeO_2_ NPs enhanced the ROS scavenging, antibacterial, and cell proliferation abilities, which greatly promoted the wound healing processes. Gong et al.^[^ ^50^ ^]^ coated cerium oxide nanorods with poly(ethylenimine) and crosslinked with benzaldehyde‐terminated F127 to prepare injectability self‐healing nanocomposite hydrogels (PVEC hydrogels) with ROS scavenging activity. They showed the biocompatible and biodegradable PVEC hydrogels significantly improved the wound healing and skin regeneration with the formation of hair follicle and adipocyte tissue. The PVEC hydrogels had excellent hydroxyl radical scavenging activity and superoxide anion inhibition rate, which is dependent on the concentration of cerium oxide nanorods. In another study, CeO_2_ NPs and an antimicrobial peptide were incorporated into a sprayable catechol‐modified hydrogel to combine ROS scavenging and antibacterial activities for wound healing.^[^ ^14^ ^]^ CeO_2_ NPs‐loaded hydrogels significantly improved the ROS scavenging ability, the rate of re‐epithelialization, collagen deposition, and wound closure compared to hydrogels containing only the antimicrobial peptide or non‐modified hydrogels. Therefore, nanoceria have great potential in ROS scavenging for the acceleration of wound healing process.

### 3.2 Carbon‐based nanomaterials

**FIGURE 4:**
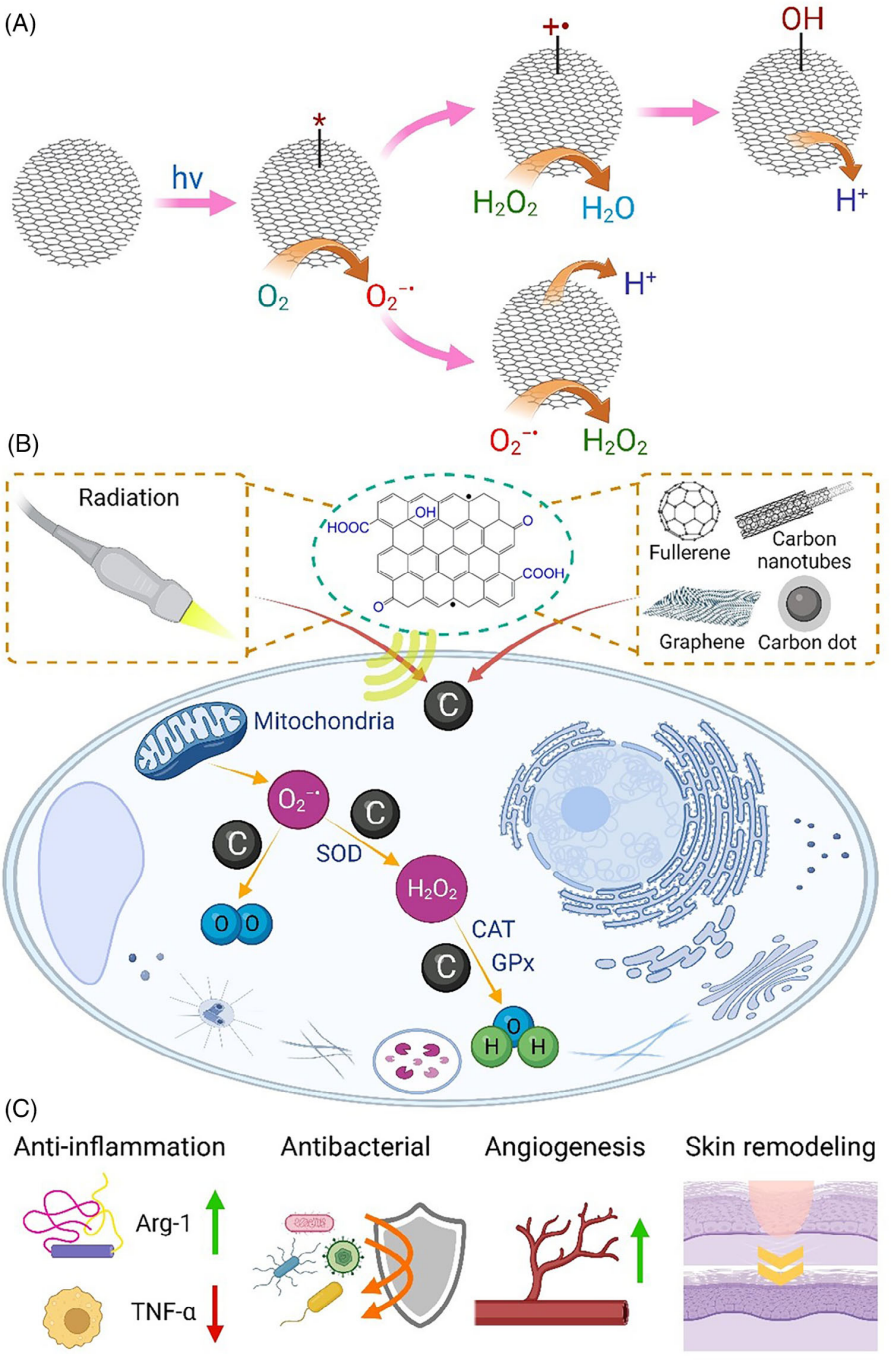
(A) The mechanism of reactive oxygen species (ROS) regeneration and scavenging by carbon‐based nanomaterials, (B) antioxidant activities of carbon‐based nanomaterials within a cell, and (C) their therapeutic effects on wound healing.

Carbon nanomaterials such as fullerenes, carbon nanotubes, and carbon quantum dots have been widely used in biomedical applications owing to their ROS scavenging ability.^[^ ^40^, ^51^, ^52^, ^53^ ^]^ ROS are adsorbed on the surface of the carbon‐based nanomaterials and the unpaired electrons are transferred to the electron deficient parts of nanomaterials to destroy the structure of ROS^[^ ^19^ ^]^ (Figure 4A). Carbon nanodots can be prepared from natural carbon sources (e.g. plants and hairs) and possess antioxidant properties (Figure 4B,C). For example, Bankoti et al.^[^ ^40^ ^]^ synthesized onion derived carbon nanodots (OCND), which were applied topically to the wound substrates to modulate ROS level and accelerate wound healing in rat models. Antioxidant efficacy of OCNDs were characterized and showed good free radical scavenging potential. They also demonstrated that OCNDs promoted wound healing in a full thickness wound in a rat model using histological analysis, comparison with control groups. Pal et al.^[^ ^54^ ^]^ developed a nanofibrous poly(caprolactone)/gelatin scaffold incorporated with CNDs synthesized from date molasses. The scaffold had excellent dermal regenerating, ROS scavenging, and fluorescent properties, leading to better in vivo wound healing in a rat model. Das et al.^[^ ^55^ ^]^ synthesized green chili extract‐derived CNDs to study wound healing kinetics. The antioxidant activity of CNDs was confirmed using in vitro and in vivo models. The wound healing potential of CNDs was observed during the histological analysis. Green and yellow carbon quantum dots (CQDs) with low toxicity, optical characteristics, and antioxidant activity were prepared by Moniruzzaman et al.^[^ ^56^ ^]^ using a three‐fold symmetric molecule, 1,3,5‐trihydroxybenzene, and incorporated in multifunctional gelatin‐methacryloyl hydrogels to enhance wound healing. Antioxidant effects of both CQDs in the human bone marrow mesenchymal stem cells and human dermal fibroblasts were evaluated. The dressing materials showed good wound healing efficiency via inducing the proliferation of fibroblasts, enhancing cell migration, alleviating inflammation, and boosting skin re‐epithelialization and collagen deposition.

### 3.3 Iron‐based nanomaterials

**FIGURE 5:**
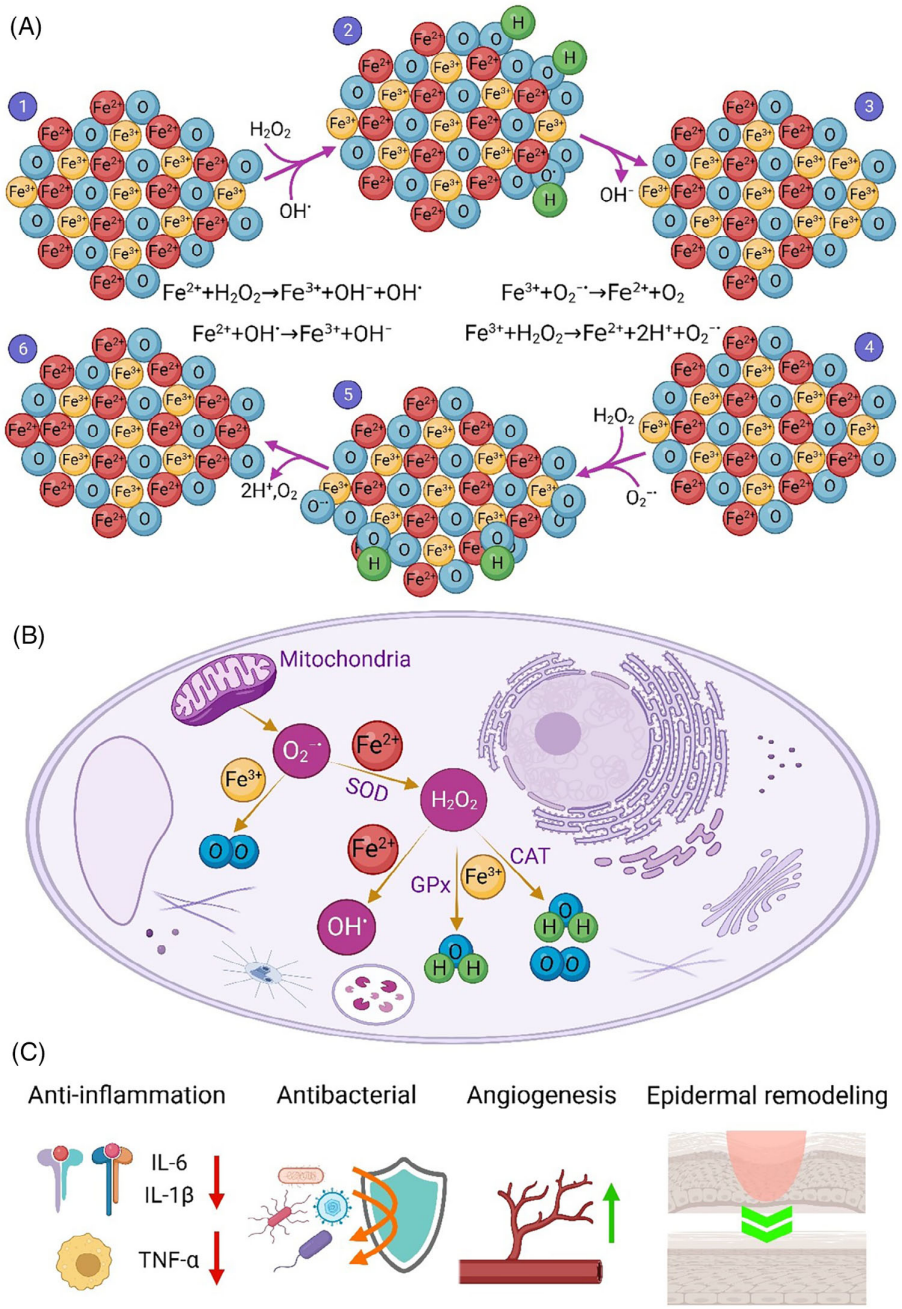
(A) Oxidation‐reduction cycle of IONzymes that are capable to regenerate and scavenge ROS, (B) antioxidant properties of IONzymes within a cell, and (C) the therapeutic effects of IONzymes toward wound healing.

Iron oxide nanoparticles (IONPs) are well‐established nanomaterials which can be engineered to have intrinsic enzyme‐like activities similar to peroxidase, CAT, and SOD.^[^ ^57^, ^58^ ^]^ Iron oxide nanoenzyme (IONzyme) based on either Fe_2_O_3_ or Fe_3_O_4_ possesses the advantages in stability, tunability of activity, and multi‐functionality due to its nanoscale property compared to natural enzymes.^[^ ^58^, ^59^ ^]^ In Figure 5A, the reversible switching between Fe^2+^ and Fe^3+^ oxidation states has been illustrated which results in ROS production and scavenging through the oxidation‐reduction cycles. The antioxidant activities of IONzymes benefits the therapeutic effects during wound healing process^[^ ^34^, ^60^, ^61^ ^]^ (Figure 5B,C). Prussian blue (PB, Fe_4_[Fe(CN)_6_]_3_.*x*H_2_O) is an iron‐based coordination compound and PB‐based nanoparticles show great enzyme mimetic properties and anti‐inflammatory activities.^[^ ^62^, ^63^, ^64^ ^]^ More recently, Sahu et al.^[^ ^65^ ^]^ synthesized PB nanozymes to investigate their healing capacity in full‐thickness mouse wound models. PB nanoenzymes showed antioxidant activities by H_2_O_2_ degradation and superoxide radicals scavenging activity in a time‐ and concentration‐dependent manner. The anti‐inflammatory effect of PB nanoenzymes was assessed using lipopolysaccharide induced RAW 264.7 macrophage cells, and the expression of pro‐inflammatory cytokines such as TNF‐α and IL‐1β was significantly reduced after PB nanozyme treatment, compared to the control samples. In addition, PB nanoenzymes treatment resulted in a higher expression of the anti‐inflammatory gene, arginase‐1 (Arg‐1), than the untreated cells. Furthermore, the topical administration of PB nanoenzymes exhibited effective collagen deposition, mature organization, keratinocyte differentiation, neovascularization, and capillary formation during wound healing process. In a similar study by Xu et al.,^[^ ^34^ ^]^ PBNPs were synthesized and incorporated into a thermosensitive hydrogel to promote diabetic wound healing via ROS scavenging and mitochondrial function restoration. PBNPs protected mitochondria from oxidative stress‐related damage and restored nuclear factor erythroid 2‐related factor 2 (NRF2)/heme oxygenase‐1 (HO‐1) pathway activity. In vivo analysis revealed that the PBNP‐incorporated hydrogels improved diabetic wound healing via reducing the inflammatory cytokines, while increasing neovascularization, and angiogenesis. In a study by Hu et al.,^[^ ^61^ ^]^ hematite (α‐Fe_2_O_3_) nanoenzyme particles were engineered by incorporating highly stable IONPs into electrospun poly(vinyl alcohol) porous scaffold to regulate ROS level in wound healing process. This electrospun hybrid nanofibrous wound dressing possesses high catalase‐like enzymatic activity to quickly convert H_2_O_2_ into O_2,_ as well as excellent wettability, water permeability, and cell proliferation in wounds. Guo et al.^[^ ^60^ ^]^ synthesized IONPs to promote peroxide‐like catalytic activity for bacteria‐infected wound therapy. They found that IONPs induced conversion of H_2_O_2_ to hydroxyl radicals to prevent bacterial infection and biofilm formation, leading to efficiently enhanced wound healing in mouse models. Du et al.^[^ ^66^ ^]^ employed IONPs coated with a shell of glucose oxidase (IONPs‐GOx) to modulate CAT and peroxide‐like activities in healing of diabetic ulcer. The IONPs‐GOx‐induced elimination of the excessive oxidative stress led to short inflammatory phase, and accelerated proliferation and tissue‐remodeling phases. Collagen deposition and the expression of CD31‐positive cells increased in wounds treated with IONPs‐GOx compared to control samples. This body of evidence suggests that IONPs possess the great potential in ROS scavenging for the treatment of pathological microenvironment‐associated diseases, especially skin regeneration.

### 3.4 Copper‐based nanomaterials

**FIGURE 6:**
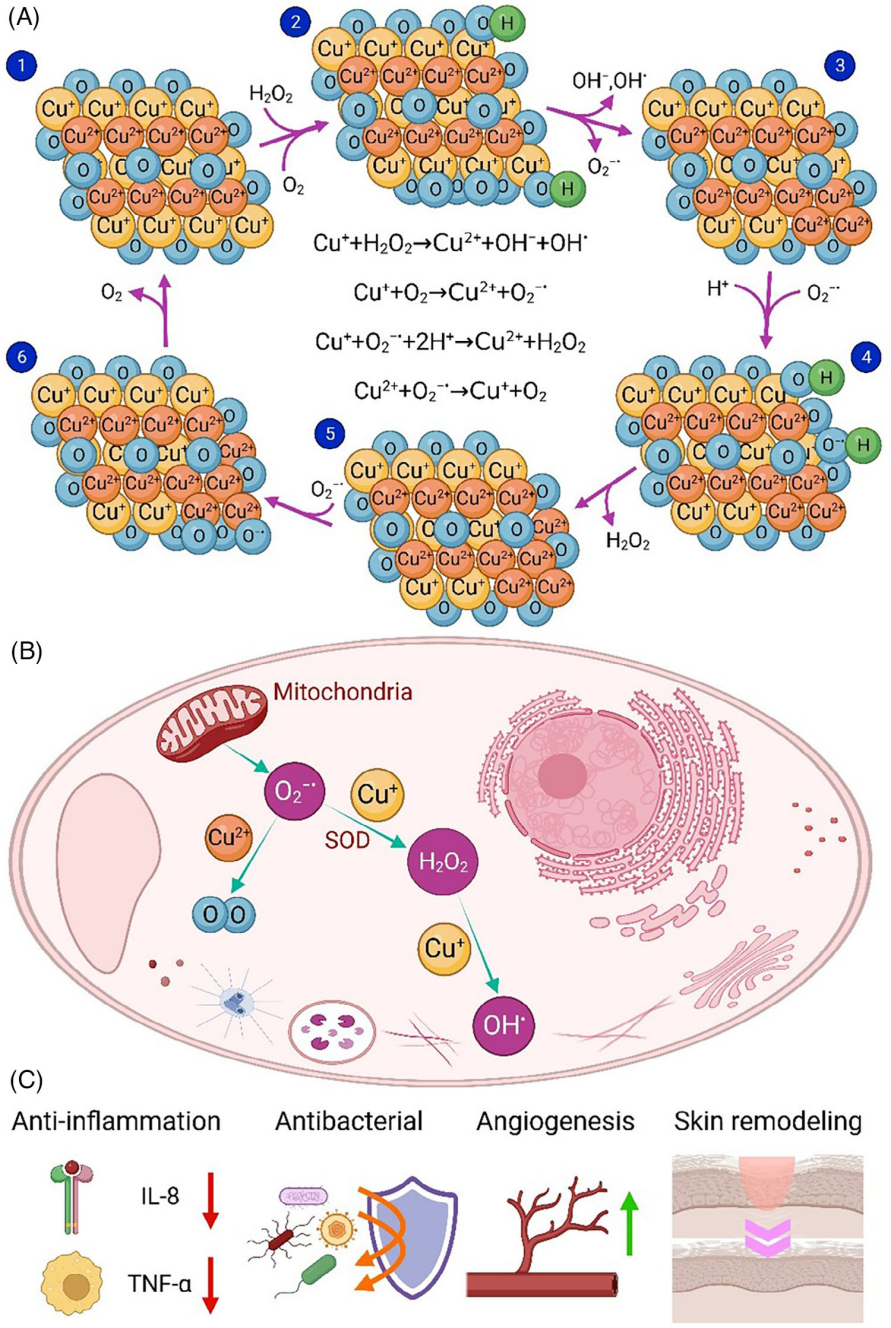
(A) Mechanism of reactive oxygen species (ROS) regeneration and scavenging in the conversion reaction of Cu^+^ to Cu^2+^, (B) antioxidant properties of CuNPs within a cell, and (C) the therapeutic effects of CuNPs in wound healing process.

Copper is an essential trace element involving in the maintenance of metabolic processes, such as hemoglobin synthesis, bone formation, and the enzyme activities.^[^ ^67^ ^]^ The intrinsic catalytic‐like activity of copper nanoparticles (CuNPs) has been indicated through 2,2′‐azino‐bis(3‐ethylbenzothiazoline‐6‐sulphonic acid) (ABTS), 2,2′‐diphenyl‐1‐picrylhydrazyl (DPPH), and H_2_O_2_ free radical scavenging assays.^[^ ^68^, ^69^ ^]^ The strong quantum confinement of electrons in ultrasmall CuNPs leads to catalytic activity and H_2_O_2_ and peroxide scavenging.^[^ ^70^ ^]^ In addition, copper oxide (Cu_2_O) can promote electron transfer reactions to inactivate H_2_O_2_ and OH^•^, thereby partially mimicking peroxidase.^[^ ^70^ ^]^ The formation and scavenging of ROS in the reversible reaction of Cu^+^ to Cu^2+^ are illustrated in Figure 6A. Moreover, fascinating features of CuNPs, including low toxicity, high stability, adhesion, and antibacterial properties, as well as antioxidant activities, make them great candidates in wound healing applications^[^ ^70^, ^71^, ^72^ ^]^ (Figure 6B,C). Liu et al.^[^ ^70^ ^]^ synthesized ultrasmall Cu_5.4_O NPs with high biocompatibility, broad ROS scavenging, and remarkable antioxidant efficiency. Cu_5.4_O NPs simultaneously possessed CAT, SOD, and glutathione peroxide enzyme‐mimicking properties. When applied to diabetic wounds, Cu_5.4_O NPs induced faster wound closure and better newly regenerated epidermis and granulation tissue formation than those in the control groups. These Cu_5.4_O NPs were further used in a heparin‐based composite hydrogel system to treat acute and diabetic wounds.^[^ ^73^ ^]^ They found that the engineered hydrogel dressing can absorb inflammatory chemokines monocyte chemoattractant protein‐1 and IL‐8, scavenge ROS to mitigate oxidative stress, and promote angiogenesis, resulting in inflammation reduction and wound recovery. Wang et al.^[^ ^72^ ^]^ synthesized copper metal organic framework nanoparticles and embedded them in carboxymethyl chitosan‐g‐glutathione/polyacrylamide hydrogels with dual effects of antibacterial and antioxidant. This hydrogel dressing significantly increased free radical scavenging rate of superoxide and hydroxyl radicals, while controlled release of copper ions improved antibacterial activity and accelerated wound healing in rat models. Therefore, CuNPs possess the potential to be used as nanoscavengers in wound healing applications.

### 3.5 Molybdenum disulfide nanomaterials

**FIGURE 7:**
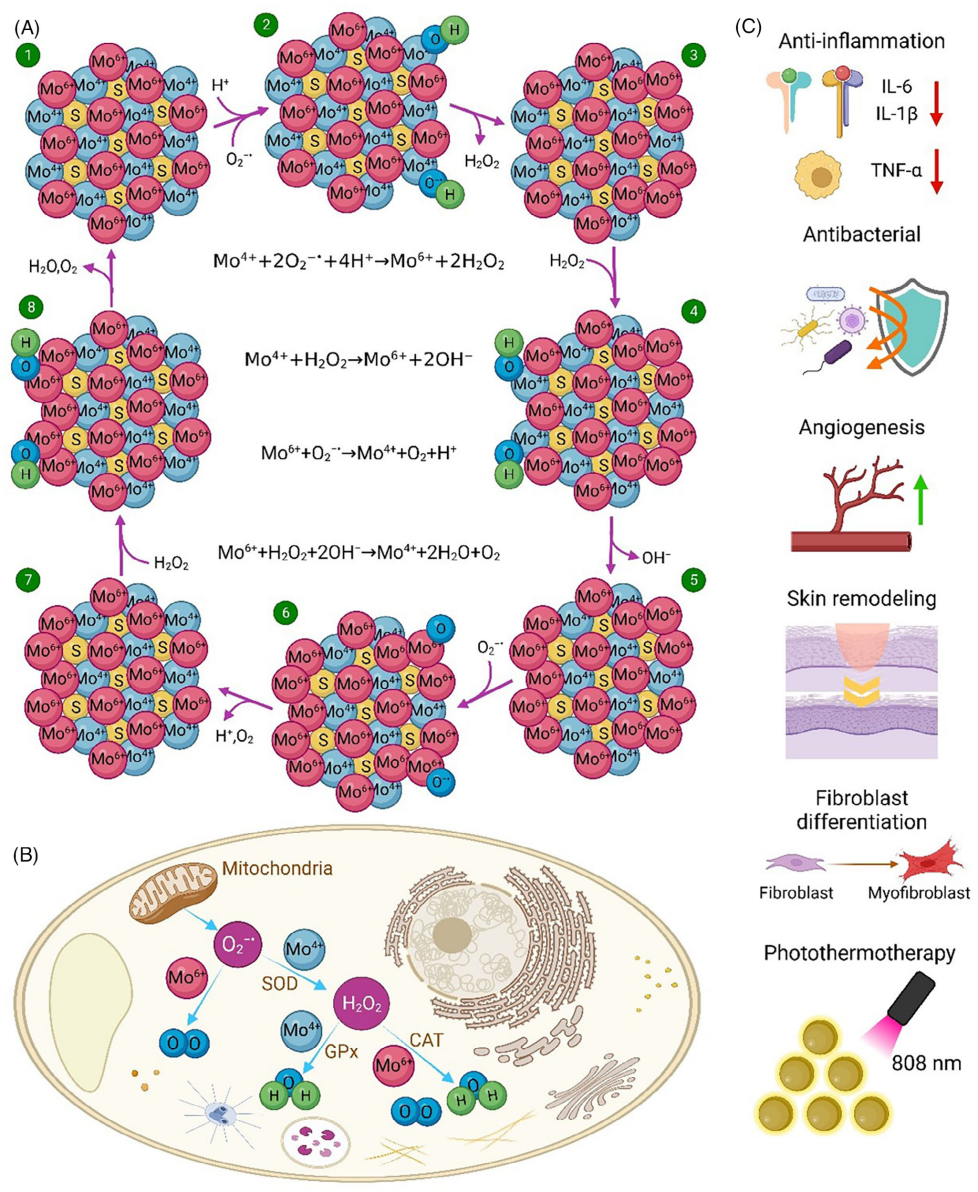
(A) Oxidation‐reduction cycle of MoS_2_‐based nanomaterials that are capable to regenerate and scavenge ROS, (B) antioxidant properties of MoS_2_‐based nanomaterials in a cell, and (C) their therapeutic effects toward wound healing.

Molybdenum (Mo) is an essential element in the biosynthesis of cofactors and take part in the formation of various enzymes in human body.^[^ ^74^ ^]^ Molybdenum disulfide (MoS_2_)‐based nanomaterials can exhibit amazing catalytic activities including peroxidase‐, CAT‐, and SOD‐like activity due to the electron transfer and valence change, making them a promising group of nanomaterials in biomedicine.^[^ ^75^, ^76^, ^77^, ^78^ ^]^ The catalytic mechanism of MoS_2_ is illustrated in Figure 7A. Currently, MoS_2_ nanostructures have the potential to be used for wound healing because of their unique mechanical and chemical properties as well as antibacterial and antioxidant activities^[^ ^78^, ^79^, ^80^, ^81^ ^]^ (Figure 7B,C). Studies show that the antioxidant activities of MoS_2_‐based nanomaterials can alleviate oxidative stress and hypoxia at the diabetic wound site. A biocompatible antibacterial system based on poly(ethylene glycol) functionalized MoS_2_ nanoflowers was developed by Yin et al.^[^ ^76^ ^]^ for wound disinfection. This synergistic antibacterial nanomaterial system efficiently catalyzed decomposition H_2_O_2_ to generate OH^•^, making bacteria more vulnerable. The MoS_2_‐enabled photothermal therapy under the 808 nm near‐infrared irradiation also led to efficient antibacterial effects and infected wound healing in mouse models. A few MoS_2_‐based hybrid materials have also been reported recently to achieve multienzyme catalytic activity for skin tissue regeneration. In a study by Li et al.,^[^ ^80^ ^]^ MoS_2_ nanosheets loaded with bovine serum albumin‐modified gold nanoparticles have been synthesized and anchored onto hydrogels to promote diabetic wound healing by reducing oxidative stress. The effect of MoS_2_ nanosheets on efficient elimination of ROS was observed in their study, leading to dramatically increased wound closure, collagen deposition, and expression level of CD31 and epidermal growth factor (EGF). MoS_2_ nanosheets were also loaded onto carbon nanotubes and incorporated in multifunctional hydrogels to kill bacteria and remove free radicals.^[^ ^81^ ^]^ The hydrogel system exhibited adhesiveness, self‐healing, and shape‐adaptivity. At the same time, it was able to boost the skin reconstruction through ROS scavenging and antibacterial action, promoting collagen deposition and angiogenesis, and controlling wound inflammation. This research suggests that MoS_2_‐based nanomaterials provide promising strategies against wound infection.

### 3.6 ROS‐scavenging polymeric nanomaterials

ROS scavenging polymers can remove excessive ROS and protect cells from inflammation.^[^ ^7^, ^82^ ^]^ Therefore, polymer‐based scavengers have received considerable attention for treatment of chronic wounds.^[^ ^15^, ^83^, ^84^, ^85^ ^]^ Poly(dopamine) (PDA) has been widely used in fabrication of wound dressing materials to scavenge ROS due to their excellent biocompatibility, tissue adhesion, and antioxidant activity.^[^ ^85^, ^86^ ^]^ The inherent antioxidative ability of PDA is attributed to abundant phenolic groups which are converted to quinones and the free radical redox equilibrium is established.^[^ ^15^, ^87^ ^]^ Liu et al.^[^ ^86^ ^]^ fabricated multifunctional poly(caprolactone) nanofibers modified with poly(l‐lysine) and PDA. Poly(l‐lysine) exhibited antibacterial activities against gram‐negative and gram‐positive bacteria, while PDA served as antioxidant agent to eliminate the excessive ROS. The PDA‐included electrospun nanofibers showed great potential as promising wound dressing materials. Poly(caprolactone)/sulfated chitosan electrospun nanofibers loaded with PDA nanoparticles and VEGF were prepared by Sheng et al.^[^ ^83^ ^]^ to promote wound healing of diabetic mice. Free radical scavenging property and biocompatibility of PDA nanoparticles on the electrospun membrane were demonstrated. In addition, enhanced wound healing through the angiogenesis and anti‐inflammatory effect of the nanofibers was observed according to the histological and immunohistochemical results, suggesting these PDA nanoparticle‐loaded nanofibers have a great potential for diabetic wound care. Fu et al.^[^ ^15^ ^]^ incorporated reduced PDA nanoparticles into oxidized dextran/chitosan hydrogels to promote antioxidant activities and antibacterial properties to accelerate wound healing. The reduced PDA nanoparticles in the hydrogels possess good free radical scavenging activity, which protected cells from oxidative damage. Fewer neutrophils and faster tissue remodeling were observed at the wound sites after the treatment of the PDA NPs incorporated polysaccharide hydrogels. In another example using PDA nanomaterials, a core‐shell hyaluronic acid microneedle patch with ferrum‐mesenchymalstem cells in the core and PDA nanoparticles in the needle tip was prepared for wound healing.^[^ ^84^ ^]^ The hyaluronic acid was degraded gradually to release PDA nanoparticles to suppress the ROS, whereas the ferrum‐mesenchymal stem cells in the core promoted proliferation, migration, and tube formation of endothelial cells. This PDA decorated microneedle system induced M2 macrophage polarization, and enhanced re‐epithelialization and collagen deposition at the wound sites. Researchers have also developed PDA nanoparticles with different structural features to optimize scavenging properties for wound treatment. Jing et al.^[^ ^85^ ^]^ prepared a 2D PDA nanosheet to act as free radical scavenger and in full‐thickness wound healing process. The histologic study shows the effectively stimulated angiogenesis and collagen deposition at the wound sites. A poly(l‐lysine)‐grafted nanocellulose/gelatin/PDA hydrogel (NCF‐EPL/GTP/PDA) was synthesized by Ren et al.^[^ ^88^ ^]^ to promote the treatment of MRSA infected wounds. The NCF‐EPL/GTP/PDA hydrogel had excellent antibacterial and antioxidant performance. Their histological results showed more continuous epidermal layer with more hair follicles and sebaceous glands, collagen deposition, and accelerated wound closure in wounds treated with NCF‐EPL/GTP/PDA hydrogels. Tannic acid, as a natural polyphenol, is composed of a central glucose molecule derivatized at its hydroxyl groups with one or more galloyl residues. Tannic acid possesses antioxidant activities, biocompatibility, and biodegradability, making it suitable for biomedical applications, particularly wound healing.^[^ ^89^, ^90^ ^]^ Tannic acid can be crosslinked to form poly(tannic acid) nanostructures. A multilayer‐structured bioactive nanoglass coated with poly(tannic acid) and poly(l‐lysine) (BGN@PTE) was developed by Wang et al.^[^ ^91^ ^]^ to enhance wound repair and angiogenesis. BGN@PTE alleviated the oxidation stress, induced the cell migration, angiogenesis, and promoted proliferation and wound treatment, suggesting they have great potential for skin tissue regeneration. You et al.^[^ ^92^ ^]^ prepared poly(tannic acid) nanorods and incorporated them into a polysaccharide matrix comprising oxidized β‐glucan and quaternized chitosan to accelerate wound healing in a diabetic rat model. Poly(tannic acid) nanorods increased ROS scavenging rate, while quaternized chitosan enhanced antibacterial activities. Thus, this multifunctional polysaccharide hydrogel dressing brought more options for the management of diabetic wounds.

## 4 NATURAL ANTIOXIDANT‐LOADED DELIVERY SYSTEM

The use of natural antioxidants (e.g. polyphenols) in order to regulate the redox balance through the modulation of ROS in wound healing applications is growing.^[^ ^23^, ^26^, ^93^ ^]^ Low water solubility, rapid clearance from the body, and limited bioavailability are the major drawbacks of some natural antioxidants, which hampered their therapeutic translation. Thus, it is crucial to develop suitable carriers to deliver the antioxidant compounds through a controlled and sustained manner to targeted lesions.^[^ ^2^, ^94^ ^]^ In this section, we summarize and discuss some of the most used natural antioxidants for wound healing process mainly by reducing oxidative stress burdens (Table 2).

**TABLE 2:** Natural compounds with antioxidant activities and their impacts on wound healing process.

Antioxidant category	Antioxidant carrier	Setting (in vitro/in vivo)	Description	Reference
Curcumin 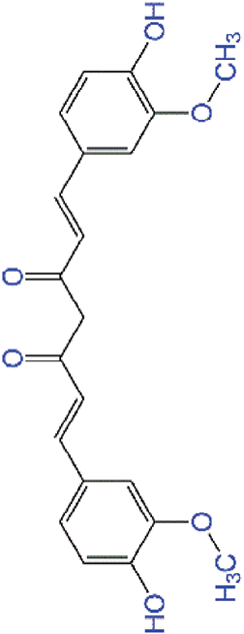	A nanofibrous scaffold containing poly(l‐lactic acid), poly(citrate siloxane) and poly(dopamine)	In vivo	Multifunctional poly(l‐lactic acid)‐poly(citrate siloxane)‐curcumin‐poly(dopamine) nanofibrous scaffolds were designed to heal bacterial‐infected wounds in mouse models. Antioxidant and anti‐inflammatory of scaffolds were measured and contributed to curcumin	[100]
	A sandwich‐like nanofibrous membrane including three layers	In vivo	A curcumin‐loaded sandwich‐like nanofibrous membrane including three layers was prepared to accelerate wound healing. The mid‐layer releases curcumin to reduce oxidative stress and inflammation.	[97]
	A poly(lactide‐*co*‐glycolide) nanofiber scaffold	In vivo	Aligned curcumin‐loaded poly(lactide‐*co*‐glycolide) nanofibers, followed by surface grafting of high density heparin were prepared to facilitate curcumin release and carry out diabetic wound healing in rat models.	[98]
	An amphiphilic alkylated‐dextran nanoparticles incorporated into hydrogels of oxidized dextran and gelatin	In vitro	An amphiphilic alkylated‐dextran nanoparticles loaded with curcumin was synthesized and incorporated into hydrogels of oxidized dextran and gelatin crosslinked through the formation of Schiff‐base reactions as wound dressing materials. CeNPs were added to the hydrogel system to increase the synergistic antioxidant activity.	[99]
	A composite nano‐fibrous material comprising poly(vinylpyrrolidone) and cerium nitrate hexahydrate	In vivo	A composite nano‐fibrous material comprising poly(vinylpyrrolidone), cerium nitrate hexahydrate, and curcumin was developed to protect skin tissues from ROS and reduce local oxidative stress for accelerated anti‐scar full thickness wound healing.	[96]
Gallic acid 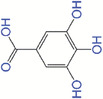	A hydrogel containing gelatin‐hydroxyphenyl propionic	In vivo	A series of injectable gelatin hydrogels with antioxidant activity was developed. Gallic acid‐conjugated gelatin was introduced to gelatin‐hydroxyphenyl propionic hydrogels to prepare effectively ROS scavenging wound dressing materials.	[21]
	A cellulose acetate nanofibrous dressing	In vitro	Gallic acid‐loaded cellulose acetate nanofibrous dressings were fabricated to show their potential for use as wound dressing materials. The antioxidant and antibacterial activities of gallic acid were determined by radical scavenging assay and disc diffusion method. Both antioxidant and antibacterial activities significantly enhanced by increasing the gallic acid concentration.	[104]
Vitamin E 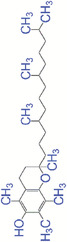	Poly(caprolactone)/gelatin electrospun mats	In vivo	Poly(caprolactone)/gelatin electrospun mats containing selenium nanoparticles and vitamin E were fabricated and their impact on wound healing was demonstrated. The highest antioxidant activity of nanofibrous scaffolds containing vitamin E was evaluated and histopathological studies exhibited significantly enhanced healing rate of skin wounds via reducing inflammatory cells and edema, and increasing neovascularization, collagen deposition, and re‐epithelialization.	[109]
	Starch nanoparticles incorporated in silk fibroin‐poly(vinylalcohol)‐Aloe vera nanofibers	In vitro	Vitamin E loaded‐starch nanoparticles were prepared and incorporated in silk fibroin‐poly(vinylalcohol)‐Aloe vera nanofibers to provide a finer control on the release of vitamin E. The cellular viability and cell‐matrix interaction were also improved by adding vitamin E, and the nanofibrous dressings have great potential for treatment of wounds.	[108]
Coenzyme Q10 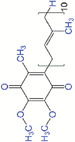	Poly(vinylalcohol) electrospun nanofibrous scaffolds	In vitro	Mupirocin, keratin, coenzyme Q10‐loaded poly(vinylalcohol) electrospun scaffolds were developed to be used as wound dressing materials. The ROS scavenging results indicated the ability of coenzyme Q10 in elimination of free radicals to prevent oxidative stress.	[112]
Epigallocatechin‐3‐gallate 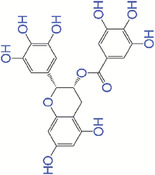	Silk fibroin hydrogels	In vivo	Epigallocatechin‐3‐gallate grafted silk fibroin hydrogels were developed to evaluate antioxidant activity of hydrogels for wound healing in rat models. The superoxide radical scavenging, hydroxyl radical scavenging, and collagenase inhibition significantly enhanced by increasing the concentration of epigallocatechin‐3‐gallate.	[119]
	A hydrogel by crosslinking 2‐hydroxyethyl methacrylamide, acrylamide, and borax	In vivo	A multifunctional hydrogel with ROS scavenging property was fabricated to protect skin‐related cells from ROS degradation and accelerate diabetic wound healing via macrophage polarization to M2 phenotype, promoting proliferation, epithelialization, collagen deposition, and neovascularization.	[120]
Quercetin 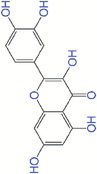	Quercetin conjugated gold nanoparticles	In vitro	Quercetin conjugated gold nanoparticles were synthesized to show its beneficial on migration of fibroblasts and in vitro wound healing. ROS scavenging capacity of quercetin conjugated gold nanoparticles was contributed to the presence of quercetin and enhanced fibroblast migration through TGF‐β mediated SMAD signaling cascade was also demonstrated.	[123]
	Poly(caprolactone) nanofibrous mat	In vivo	A poly(caprolactone) nanofibrous mat loaded with ciprofloxacin hydrochloride and quercetin was developed to suppress bacterial infections and oxidative damages during wound healing in rat models. The nanofibers containing quercetin showed the best ROS scavenging rate.	[124]
Tannic acid 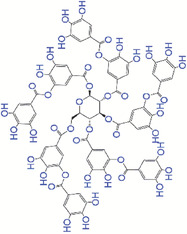	Zwitterionic poly(sulfobetaine methacrylate) hydrogels	In vivo	Attributed to the antioxidant activity of tannic acid, the hydrogels showed rapid radical scavenging ability. Promoted healing of diabetic wounds in mouse models was observed in wounds treated with tannic acid‐reinforced zwitterionic hydrogels.	[130]
	Carboxymethyl chitosan/tannic acid hydrogels	In vivo	Hydrogels were fabricated with antibacterial, antioxidant, as well as quick hemostasis capabilities. In vivo analysis exhibited the potential of the hydrogels in reducing inflammation, and increasing wound closure, re‐epithelialization, and collagen deposition.	[132]
	Chitin/polyethylene glycol diglycidyl ether hydrogels	In vivo	Tannic acid was used as a crosslinking agent to prepare multifunctional hydrogels with antibacterial, antioxidant, and hemostatic properties to promote wound healing in rat models by regulating inflammatory response, collagen deposition, and blood vessel formation.	[131]
	A bilayer hydrogel consists of the bottom layer of hyaluronic acid, poly(vinyl alcohol), and poly(ethylene glycol) and the top layer of carboxylated chitosan, poly(vinyl alcohol), and poly(ethylene glycol)	In vivo	A bilayer hydrogel was prepared and modified with tannic acid (TA@bilayer). ROS scavenging activities of TA@bilayer hydrogels significantly increased compared to bilayer hydrogels without tannic acid. Accelerated wound healing via reducing TNF‐α level, and facilitating the expression of VEGF and collagen deposition was observed in wounds treated with TA@bilayer.	[129]
	A multifunctional methacrylated chitosan/methacrylated silk hydrogel	In vivo	Tannic acid improved antioxidant activity of the hydrogels. Tannic acid‐reinforced hydrogels promoted full‐thickness wound treatment with completely recovered the epidermal layer and the formation of hair follicles.	[128]

### 4.1 Curcumin

Curcumin is a natural polyphenolic molecule extracted from turmeric plant with anti‐inflammatory, antibacterial, and antioxidant properties. It is reported that curcumin can improve wound healing via granulation tissue formation, collagen deposition, and tissue reconstruction.^[^ ^9^, ^95^, ^96^ ^]^ Many novel dressing formulations have been engineered to overcome its bioavailability, solubility, and stability issues. For example, a curcumin‐loaded sandwich‐like nanofibrous membrane was prepared by Chen et al.^[^ ^97^ ^]^ using electrospinning method as wound dressing for accelerated wound healing. The hemostatic nanofibrous membrane consisting of gelatin, chitosan, and poly(caprolactone) was able to stop bleeding, absorb exudates, and keep the wound moist, while curcumin‐loaded membrane reduced oxidative stress and inflammation. The dressing also had an AgNPs‐contained antibacterial membrane to avoid wound infection. They found curcumin significantly increased the antioxidant activity of the fabricated wound dressings, reduced inflammation response and accelerated epidermal regeneration and collagen deposition. Liao et al.^[^ ^98^ ^]^ prepared heparin‐grafted aligned curcumin‐loaded poly(lactide‐*co*‐glycolide) nanofiber membranes (PCH NFMs) to facilitate curcumin release and diabetic wound healing in rat models. Benefited from the antioxidant activity of curcumin, PCH NFMs improved wound closure and skin tissue regeneration by relieving ROS and inflammatory cascade while enhancing angiogenesis and collagen deposition. In addition, researchers have combined curcumin with other ROS scavenging agents to enhance their antioxidant and anti‐inflammatory effects. A composite nano‐fibrous material comprising poly(vinylpyrrolidone), cerium nitrate hexahydrate, and curcumin was developed by Pandey et al.^[^ ^96^ ^]^ to reduce local oxidative stress and accelerate anti‐scar full thickness wound healing. Their data suggested synergistic effect of curcumin and Ce^3+^ on scar recovery by inhibiting microbial infection and oxidative shock. In a study by Andrabi et al.,^[^ ^99^ ^]^ a gelatin and oxidized dextran‐based nano‐hybrid hydrogel was synthesized as wound dressing materials and curcumin and CeNPs were incorporated into the hydrogels. They observed the synergistic antioxidant and anti‐inflammatory activity of curcumin and CeNPs after applying the hybrid dressing. Xi et al.^[^ ^100^ ^]^ designed a multifunctional poly(l‐lactic acid)‐poly(citrate siloxane)‐curcumin‐poly(dopamine) nanofibrous scaffolds to heal bacterial‐infected wounds in a skin tumor mouse model. Curcumin in the scaffolds showed excellent antibacterial and antioxidative activities, resulting in enhanced skin regeneration including epidermis thickness and cell density of epidermis near to normal skin.

### 4.2 Gallic acid

Gallic acid is also a natural polyphenol found in most of plants. It has attracted great attentions in chronic skin diseases due to its antioxidant, anti‐inflammatory, antibacterial, and analgesic properties.^[^ ^101^, ^102^, ^103^ ^]^ Gallic acid‐loaded cellulose acetate nanofibrous dressings were fabricated by Wutticharoenmongkol et al.^[^ ^104^ ^]^ to show their potential in wound healing. The antioxidant and antibacterial activities of gallic acid were confirmed, whereas the therapeutic potential was not evaluated using in vitro and in vivo models. Thi et al.^[^ ^21^ ^]^ developed gallic acid‐conjugated gelatin‐hydroxyphenyl propionic hydrogels (GH/GGA) as an injectable wound healing material. Their data suggested that the GH/GGA hydrogels efficiently accelerated wound healing by suppressing oxidative damage and promoting hair follicle formation, neovascularization, and the alignment of collagen fiber.

### 4.3 Vitamin E

Known as an essential micronutrient, vitamin E is an antioxidant with many health benefits. Vitamin E (α‐tocopherol) is a lipid‐soluble antioxidant found in virtually all cell membrane, particularly mitochondrial membrane.^[^ ^105^, ^106^, ^107^ ^]^ Vitamin E showed positive roles for skin care due to their antioxidant, anti‐inflammatory, and scar‐preventing properties. Vitamin E loaded‐starch nanoparticles were prepared and incorporated in silk fibroin‐poly(vinylalcohol)‐Aloe vera nanofibers to provide a finer control on the release of vitamin E.^[^ ^108^ ^]^ It was demonstrated that vitamin E in the nanocomposite dressing greatly decreased oxidative stress and improved fibroblast proliferation and cell‐matrix interaction, indicating the vitamin E‐loaded nanofibrous dressings have great potential for treatment of wounds. Poly(caprolactone)/gelatin electrospun mats containing selenium nanoparticles and vitamin E (PCL/GEL/Se NPs/VE) were fabricated and their impact on wound healing was demonstrated.^[^ ^109^ ^]^ The PCL/GEL/Se NPs/VE scaffolds had the highest antioxidant activity, and improved skin wound healing based on the histopathological observation including complete re‐epithelialization, low level of edema and inflammatory cells and high level of oriented collagens.

### 4.4 Coenzyme Q10

Coenzyme Q10 is a potent antioxidant which can effectively enhance wound healing and treatment of injuries.^[^ ^110^, ^111^, ^112^ ^]^ Coenzyme Q10 can protect membrane proteins and DNA from the oxidative damages and improve mitochondrial functions and energy metabolism efficiency.^[^ ^113^, ^114^, ^115^ ^]^ Amajuoyi et al.^[^ ^112^ ^]^ fabricated mupirocin, keratin, coenzyme Q10‐loaded poly(vinylalcohol) electrospun scaffolds as wound dressing. The ROS scavenging results indicated the ability of coenzyme Q10 in elimination of free radicals to prevent oxidative stress.

### 4.5 Epigallocatechin‐3‐gallate

Epigallocatechin‐3‐gallate (EGCG) is a major polyphenolic compound present in green tea and mainly ascribed to its antioxidant and anti‐inflammatory action.^[^ ^116^, ^117^, ^118^ ^]^ However, poor bioavailability, rapid metabolism, and some unwanted effects have hindered their clinical application.^[^ ^118^ ^]^ Therefore, preparation of suitable carriers for controlled delivery of EGCG is vital. More recently, EGCG‐grafted silk fibroin hydrogels were developed by Lee et al.^[^ ^119^ ^]^ for wound healing in a rat model of full thickness skin defect. Introducing EGCG in the silk fibroin hydrogels enhanced their superoxide radical and hydroxyl radical scavenging abilities, which significantly facilitated wound closure and collagen deposition. Jia et al.^[^ ^120^ ^]^ fabricated a multifunctional hydrogel with ROS scavenging property by crosslinking EGCG, 2‐hydroxyethyl methacrylamide, acrylamide, and borax. This EGCG‐loaded hydrogel scavenged the accumulated ROS and reduced ROS‐induced cell death. In vivo results showed that the EGCG‐loaded hydrogel accelerated diabetic wound healing by activating macrophage polarization to M2 phenotype, promoting proliferation, epithelialization, collagen deposition, and neovascularization.

### 4.6 Quercetin

Quercetin is a natural flavonoid with antioxidant activity that is widely extracted from fruit and vegetable resources.^[^ ^2^, ^121^, ^122^ ^]^ Taking advantages of the strong antioxidant, anti‐inflammatory, antimicrobial, and angiogenic properties of quercetin, quercetin‐containing dressings are promising approaches for wound healing applications. Quercetin‐conjugated gold nanoparticles were synthesized by Madhyastha et al.^[^ ^123^ ^]^ and its beneficial effects on fibroblasts and wound healing were demonstrated. Quercetin‐conjugated gold nanoparticles had superior ROS scavenging capacity due to the presence of quercetin and enhanced fibroblast migration through TGF‐β mediated SMAD signaling cascade. Ajmal et al.^[^ ^124^ ^]^ developed a poly(caprolactone) electrospun nanofibrous membrane loaded with ciprofloxacin hydrochloride and quercetin to suppress bacterial infections and oxidative damages during wound healing in a rat model. The nanofibers containing quercetin effectively restored the SOD, CAT, and hydroxyproline level at the wound sites and showed the best ROS scavenging rate compared with other groups.

### 4.7 Tannic acid

Tannic acid as a naturally polyphenol has attracted attention wound healing over the past decade due to its unique properties including antimicrobial properties, antioxidant and homeostatic activities, and biocompatibility.^[^ ^125^, ^126^, ^127^ ^]^ It can be easily incorporated in hydrogels through hydrogen bonding and ion coordination as wound healing dressing. For instance, He et al.^[^ ^128^ ^]^ developed a multifunctional tannic acid‐reinforced methacrylated chitosan/methacrylated silk hydrogel. Tannic acid not only improved the mechanical properties of the hydrogel, but also boosted their antioxidant and antibacterial activity. Their animal work showed that this tannic acid‐reinforced hydrogel had superior wound recovery performance in a full‐thickness skin defect model. Li et al.^[^ ^129^ ^]^ prepared a tannic acid‐based bilayer hydrogel (TA@bilayer). Tannic acid significantly increased ROS scavenging activities of TA@bilayer hydrogels compared to bilayer hydrogels without tannic acid. TA@bilayer also improved wound healing process by preventing infection, thickening granulation tissue, and increasing collagen deposition. Tannic acid was incorporated in zwitterionic poly(sulfobetaine methacrylate) hydrogels to reinforce mechanical properties and increase adhesion to skin tissue for diabetic wound treatment by Fang et al.^[^ ^130^ ^]^ The hydrogel showed rapid radical scavenging ability and strong bactericidal efficacy due to the presence of tannic acid. They also observed positive effects of the hydrogel on difficult‐to‐heal diabetic wounds. Cao et al.^[^ ^131^ ^]^ prepare multifunctional chitin/polyethylene glycol diglycidyl ether hydrogels with tannic acid through chemical‐ and physical‐crosslinking strategies. This hydrogel showed antibacterial, antioxidant, and hemostatic properties and promoted wound healing in a full‐thickness skin defect rat model by regulating inflammatory response, collagen deposition, and blood vessel formation. Zhou et al.^[^ ^132^ ^]^ fabricated carboxymethyl chitosan/tannic acid hydrogels with antibacterial, antioxidant, as well as quick hemostasis capabilities. The in vivo results demonstrated that the hydrogels reduced inflammation, while increased wound closure, re‐epithelialization, and collagen deposition.

## 5 CONCLUSIONS AND PROSPECTS

Redox balance plays a critical role in different molecular and cellular activities and signaling pathways during wound healing and skin tissue regeneration. However, excessive ROS production under diseased conditions, particularly chronic wounds, prolongs inflammatory response and causes oxidative damages to subcellular components such as DNA and proteins, resulting in impaired regeneration process. Therefore, dynamic modulation of ROS level is essential for efficient wound treatment. Although natural antioxidants showed some positive effects on suppressing oxidative stress during wound treatment, their low bioavailability and solubility, rapid clearance from the lesions, and side effects limited the clinical translation of antioxidant therapy. Novel ROS scavenging strategies including organic/inorganic nanoparticles, ROS responsive polymers, and antioxidant‐loaded dressings have been developed to restore redox hemostasis in skin regeneration. Organic/inorganic nanoparticles eliminate excessive ROS through redox reactions depending on their valence states. However, there are great concerns around their scavenging duration and capability, speed of removal under physiological conditions, and the toxicity of certain ROS nanoscavengers. Hence, it is important to optimize nanoscavengers in order to provide ideal therapeutic antioxidant activities at the wound sites relevant to the unique circumstances of injuries. When it comes to ROS responsive polymers, the scaffolds/dressings can be designed to release antioxidants sustainably and reduce ROS around wound lesion. They can also protect nanoparticles or antioxidant molecules from degradation and overcome the limitations of natural antioxidants including poor solubility, low bioavailability, poor permeability, and instability. Nevertheless, more studies are required to evaluate ROS scavenging capacity of dressings based on their structure, degradation and release profiles in order to achieve optimal wound healing in the physiological and pathological conditions. The performance issues and long‐term bioavailability studies will be the research focus in the future. In addition, the hybrid systems offer a new path forward for nanomedicine‐based antioxidant therapies for wound healing. For example, cell‐derived exosomes have the great potential to be used as novel nano‐carriers for natural antioxidants or nanoenzymes for wound repair because they can avoid the rapid clearance by the immune system, reduce biotoxicity, and remove the unnecessary complexity in the clinical applications of cell‐mediated drug delivery systems.^[^ ^5^, ^133^ ^]^

It is extremely crucial to assess the pre‐clinical and clinical development of ROS scavenging nanomaterials. Currently there are fewer clinical studies available and nearly none of the clinical trials have been completed or published. The lack of human data hindered the clinical application and commercialization of this novel nanotherapeutic technology. Therefore, systematic investigations are required to enable clinical success of ROS nanoscavenger therapies for skin regeneration.

## AUTHOR CONTRIBUTIONS

Tianqing Liu conceptualized, guided, and edited the manuscript. Alireza Joorabloo prepared the original manuscript. Both the authors read and approved the final manuscript.

## CONFLICTS OF INTEREST STATEMENT

The authors declare no conflicts of interest.
